# The Roles of the *Saccharomyces cerevisiae* RecQ Helicase *SGS1* in Meiotic Genome Surveillance

**DOI:** 10.1371/journal.pone.0015380

**Published:** 2010-11-09

**Authors:** Amit Dipak Amin, Alexandre B. H. Chaix, Robert P. Mason, Richard M. Badge, Rhona H. Borts

**Affiliations:** Department of Genetics, University of Leicester, Leicester, United Kingdom; National Cancer Institute, United States of America

## Abstract

**Background:**

The *Saccharomyces cerevisiae* RecQ helicase Sgs1 is essential for mitotic and meiotic genome stability. The stage at which Sgs1 acts during meiosis is subject to debate. Cytological experiments showed that a deletion of *SGS1* leads to an increase in synapsis initiation complexes and axial associations leading to the proposal that it has an early role in unwinding surplus strand invasion events. Physical studies of recombination intermediates implicate it in the dissolution of double Holliday junctions between sister chromatids.

**Methodology/Principal Findings:**

In this work, we observed an increase in meiotic recombination between diverged sequences (homeologous recombination) and an increase in unequal sister chromatid events when *SGS1* is deleted. The first of these observations is most consistent with an early role of Sgs1 in unwinding inappropriate strand invasion events while the second is consistent with unwinding or dissolution of recombination intermediates in an Mlh1- and Top3-dependent manner. We also provide data that suggest that Sgs1 is involved in the rejection of ‘second strand capture’ when sequence divergence is present. Finally, we have identified a novel class of tetrads where non-sister spores (pairs of spores where each contains a centromere marker from a different parent) are inviable. We propose a model for this unusual pattern of viability based on the inability of *sgs1* mutants to untangle intertwined chromosomes. Our data suggest that this role of Sgs1 is not dependent on its interaction with Top3. We propose that in the absence of *SGS1* chromosomes may sometimes remain entangled at the end of pre-meiotic replication. This, combined with reciprocal crossing over, could lead to physical destruction of the recombined and entangled chromosomes. We hypothesise that Sgs1, acting in concert with the topoisomerase Top2, resolves these structures.

**Conclusions:**

This work provides evidence that Sgs1 interacts with various partner proteins to maintain genome stability throughout meiosis.

## Introduction

### Meiotic Recombination

During meiosis, the process of homologous recombination is critical for ensuring accurate chromosome segregation and in generating genetic diversity. Homologous recombination is initiated by a double-strand break (DSB) catalysed by Spo11 [1]. Following the formation of the DSB, 5′ strand resection generates 3′ single-stranded overhangs that are then able to invade the homolog [2]. Strand invasion is facilitated by the strand-exchange proteins Rad51 and Dmc1 [3] and leads to the formation of a Single End Invasion (SEI) structure [4].

When breaks are repaired via the crossover pathway, the second single-stranded end is captured by the D-loop following invasion and DNA synthesis. Finally, ligation leads to formation of a double Holliday junction (dHJ) [4], [5]. Resolution of the dHJ yields a crossover [6]. This pathway is promoted by the ZMM proteins (Mer3, Msh4, Msh5, Zip1, Zip2, Zip3 and Zip4), as well as Exo1, Mlh1 and Mlh3 (reviewed in [7]). Non-crossover products arise from the Synthesis Dependent Strand Annealing (SDSA) pathway [8]. The invading strand is not captured as in the crossover pathway, but is instead displaced. This is followed by strand annealing to complementary sequences on the second DSB end, and DNA synthesis, culminating in the formation of a non-crossover product.

**Figure 1:**
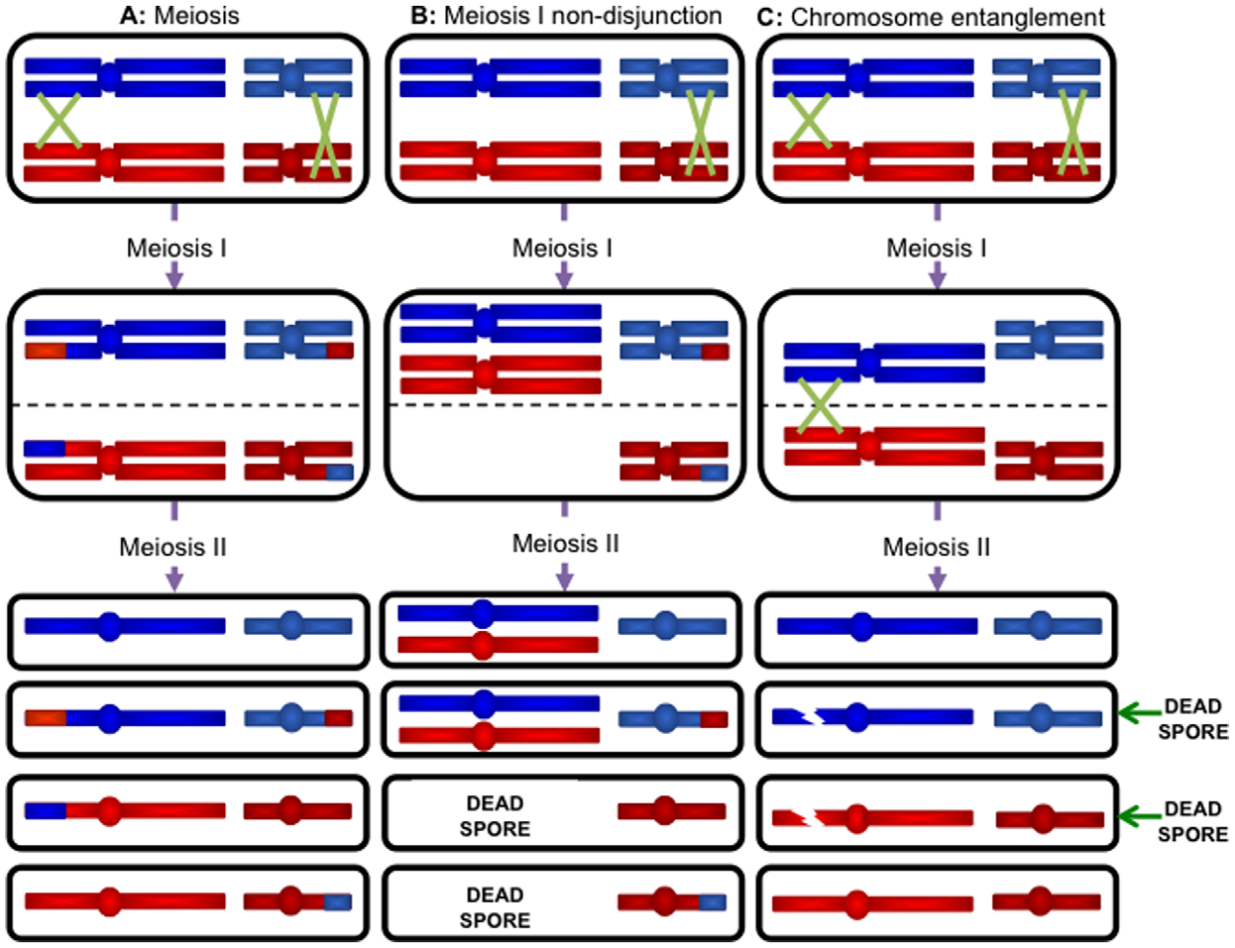
Mis-segregation events during meiosis. During meiosis, crossing over ensures the accurate segregation of homologs at meiosis I. Sister chromatids separate at meiosis II. In yeast, all four meiotic products are recovered as viable spores (**A**). The absence of crossovers may lead to both homologs becoming pulled towards the same pole at meiosis I. Meiosis I non-disjunction leads to two disomic spores (**B**). The inability to resolve entangled chromosomes can lead to chromosome breakage. A centromere marker on a pair of normally segregating chromosomes can be used to identify the sister and non-sister spores. In the case of meiosis I non-disjunction, these are sister spores (**B**). In **Figure 1C** the inability to resolve the crossover leads to the two viable spores being non-sisters (**C**).

Crossovers lead to the establishment of chiasmata, which provide the physical connections during meiotic prophase that promote the accurate segregation of homologous chromosomes (**Figure 1A**). Therefore, the absence of crossovers leads to the mis-segregation defect known as meiosis I non-disjunction (**Figure 1B**) [9]. Another class of segregation defect, known as precocious separation of sister chromatids (PSSC), is thought to arise from hyper-recombination at centromeres [10].

### Homeologous recombination and its suppression

The process of homologous recombination allows the transfer of genetic information between nearly identical stretches of DNA. In contrast, homeologous recombination is the transfer of genetic information between sequences that are diverged. In order to maintain the integrity of chromosomes, and ultimately the genome, crossing over between diverged sequences must be regulated. Interactions between diverged tandem and/or interspersed repeat sequences, such as the abundant Alu family found in primates, must be suppressed in order to prevent translocations, deletions or inversions [11]–[14]. While chromosomal rearrangements of this type may be important for driving evolution, adaptation and speciation, they are also responsible for causing disease [15], [16]. Thus despite potential evolutionary advantages, preventing recombination between diverged repeats is important [17].

Several studies have shown that the prokaryotic and eukaryotic mismatch repair (MMR) systems (reviewed in [18]) are involved in the regulation of homeologous recombination. One of the earliest of these studies [19] demonstrated that mutation of *mutS*, *mutL*, *mutH* or *mutU* leads to an increase in recombination in conjugational crosses between *E. coli* and *S. typhimurium* of up to 1000-fold. This led Rayssiguier *et al*. to suggest that the MMR system enforces a barrier to inter-species recombination [19].

The eukaryotic MMR proteins Msh2, Msh3, Msh6, Mlh1 and Pms1 also enforce a barrier to both mitotic and meiotic homeologous recombination in yeasts. Several studies in *S. cer*evisiae have shown that mutations of mismatch repair genes lead to an increase in mitotic homeologous recombination [20]–[22]. Hunter *et al*. [23] showed that mismatch repair proteins also play an important role in preventing meiotic homeologous recombination. They demonstrated that whie an inter-specific hybrid between the sibling species *S. cerevisiae* and *S. paradoxus* was able to perfectly grow mitotically, it had severe meiotic defects. Only 1% of the spore colonies were viable and they exhibited a slow growth phenotype due to aneuploidy [23]. This aneuploidy was attributed to increased meiosis I non-disjunction as a consequence of a decrease in the rates of recombination [23]. However, mutation of *PMS1* or *MSH2* leads to significant increase in the rate of recombination, which is accompanied by a decrease in the rates of meiosis I non-disjunction and an improvement in overall spore viability [23]. These observations led them to propose that the MMR system is involved in the assessment of the degree of divergence when heteroduplex DNA is formed.

These data were confirmed and extended by Chambers *et al*. [24], utilizing a partial hybrid strain in which the chromosome III from *S. cerevisiae* was replaced by chromosome III from *S. paradoxus*. This system facilitates the stuffy of the effects of sequence divergence as it does not cause massive aneuploidy. The improved viability allowed Chambers *et al*. [24] to notice an increase in the number of three viable spore tetrads in the partial hybrid strain. By inferring the genotype of the dead spore using the *1^st^ Law of Mendel* (which defines segregation), they noted that the majority of dead spores would have contained a recombinant chromosome III. The observation led to the proposal that it was the attempt to carry out recombination between diverged sequences that resulted in spore death [24]. The authors hypothesised that if one side of the DSB successfully invaded, but double Holliday junction formation failed at the strand capture stage [4] due to sequence divergence, the result would be a ‘half-crossover’ with the unrepaired reciprocal product leading to death of the spore containing it [24]. This hypothesis was confirmed by the observation that deletion of *MSH2* or *PMS1* abolished this phenotype.

### Sgs1 is the *S. cerevisiae* homolog of the RecQ helicase family

The RecQ helicase family has been implicated in maintaining the fidelity of both mitotic and meiotic recombination. Their importance is indicated by the observation that mutations in three of the five human orthologs have been associated in cancer predisposition syndromes (reviewed in [25], [26]). The *S. cerevisiae* ortholog, Sgs1, is involved in the DNA damage response during mitotic DNA replication as *sgs1* cells are sensitive to the DNA damaging agents methyl methanesulfonate (MMS) and hydroxyurea (HU) [27]–[30] and *sgs1* diploids also display moderate levels of sensitivity to UV and X-ray irradiation [31]–[33].

**Figure 2:**
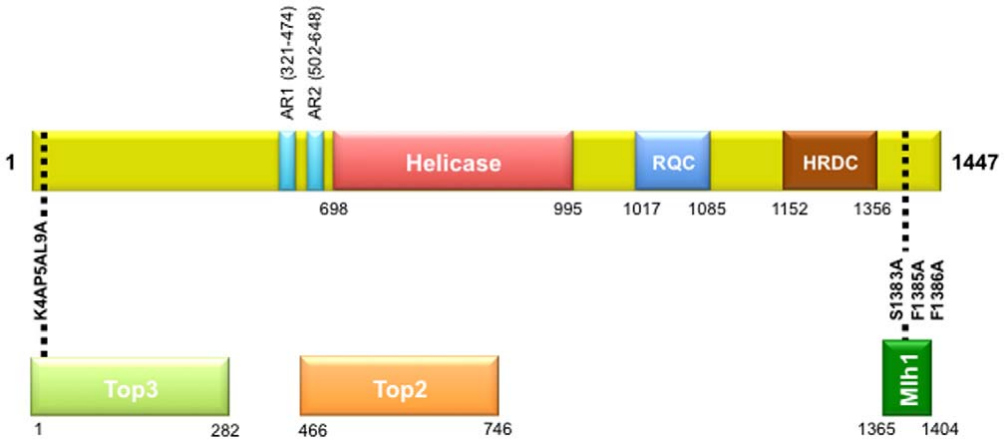
Structural and functional domains of Sgs1. The interacting domains of *SGS1* (shown with amino acid coordinates) highlighting the point mutations used in this study that disrupt the Top3-interacting domain of Sgs1 (*sgs1-top3-id*) and disrupt the Mlh1-interacting domain of Sgs1 (*sgs1-mlh1-id*). The RecQ Conserved (RQC) domain facilitates protein-protein interactions. The Helicase-and-RNaseD-C-terminal (HRDC) domain is required for DNA binding. The helicase domain facilitates the unwinding of recombination intermediates. Sgs1 also interacts with Top2 and the Top2-interacting domain of Sgs1 overlaps the helicase domain and two highly acidic regions (AR) found in the protein (as described in the *Introduction*).

Replication forks may stall due to damage on either the leading or lagging strand. Sgs1, through its association with Top3 and Rmi1, act in restarting stalled replication forks [34]–[36]. The branch migration activity of Sgs1 is proposed to form a Holliday junction-like intermediate that is dissolved by Top3 and Rmi1 to form a non-crossover product [37]–[41]. The role of Sgs1 in this process is mediated by its 3′-to-5′ helicase domain [42]–[44], while the N-terminus of Sgs1 has been shown to interact with Top3, with amino acids 4, 5 and 9 of Sgs1 being most important for this interaction [45]–[49] (**Figure 2**).

Sgs1 is also able to interact with the topoisomerase Top2 [50], [51] (**Figure 2**). This interaction occurs in regions that overlap both the acidic regions (ARs), and the helicase domain of Sgs1 (**Figure 2**) [52]. Top2 is the major mitotic post-replication decatenase [53]. In its absence, chromosomes mis-segregate, leading to both chromosome loss and disomy. Watt *et al*. [51] demonstrated that Sgs1 acts in the same pathway as Top2, suggesting that the interaction between Sgs1 and Top2 is important for the decatenation of sister chromosomes. Watt *et al*. [51] also noted aneuploidy during meiosis which they attributed to a similar failure to decatenate sister chromosomes.

Sgs1 also negatively regulates crossovers during meiosis. Deletion of *SGS1* leads to an increase in closely spaced crossovers without an apparent increase in the number of gene conversions or non-crossovers [54]–[56]. The data therefore implicate Sgs1 as an anti-crossover factor, whose actions are opposed by the pro-crossover activities of the ZMM proteins [54]–[56]. Sgs1 acts to specifically inhibit the formation of closely spaced inter-sister and multi-chromatid crossovers [55], [57]. Failure to carry out this inhibition may be detrimental to the cell, as their presence may perturb chromosome segregation [58].

### Sgs1 acts in the suppression of mitotic homeologous recombination

As discussed above, several studies implicated heteroduplex rejection by MMR proteins in the suppression of mitotic homeologous recombination [19]–[22], [59], [60] and suggested that heteroduplex rejection might require a helicase [59]–[61]. Consistent with this, *SGS1* mutations were found in screens for elevated mitotic homeologous recombination where it was shown to act in the same pathway to suppress mitotic homeologous recombination as the MMR genes *MSH2* and *MLH1* [14], [62]. An increase in the rate of mitotic homeologous recombination was also seen for a truncation mutation that deleted the C-terminal 200 amino acids of Sgs1, which contains the Mlh1 interacting domain [63], [64], suggesting that the interaction with Mlh1 may be important for the suppression of mitotic homeologous recombination [62]. Studies by Myung *et al*. [65] and Putnam *et al*. [66] also implicate the topoisomerase Top3 in suppressing rearrangements between ectopic copes of diverged sequences. These data are consistent with models in which Sgs1 unwinds homeologous intermediates and acts to dissolve crossovers between inappropriate substrates. Recently, a second helicase, Mph1, has been shown to be partially redundant to Sgs1 in the suppression of mitotic homeologous recombination [67].

The aim of this study was to further elucidate the mechanism by which homeologous recombination is suppressed in meiosis. Preliminary data obtained from a screen to identify genes whose mutation leads to increased homeologous recombination suggested Sgs1 might be involved in this process [68]. The data presented here indicate that Sgs1 acts to suppress recombination between diverged sequences at both the single-end invasion stage and at the strand capture stage. Also, in some *SGS1* mutant strains, we find an elevated frequency of an unusual class of two viable spore tetrads containing non-sister spores (as shown in **Figure 1C**). We present a model showing how these might arise due to a failure to decatenate sister chromatids after pre-meiotic DNA replication. This work therefore highlights the importance of Sgs1 during a number of different stages of meiosis.

## Results and Discussion

To investigate the role of Sgs1 in the suppression of meiotic homeologous recombination and sister chromatid exchange, we created a variety of *SGS1* mutations in the partial hybrid strain in which chromosome III from *S. cerevisiae* was replaced with chromosome III from *S. paradoxus* [24] (see **Figure 2** and *Methods and M*aterials for description, **Table S1** for haploid strain list and **Table S2** for diploid strain list). We also replaced the endogenous promoter of *SGS1* with the promoter of the *CLB2* gene (*pCLB2-SGS1*) to create a meiotic null of Sgs1 [54], [57], [58], [69] (as described in *Methods and Materials*). In order to measure unequal sister chromatid exchange we inserted a reporter construct on *S. cerevisiae* chromosome III, as described in *Methods and Materials*.

### Sgs1 acts in the suppression of meiotic homeologous recombination

**Figure 3:**
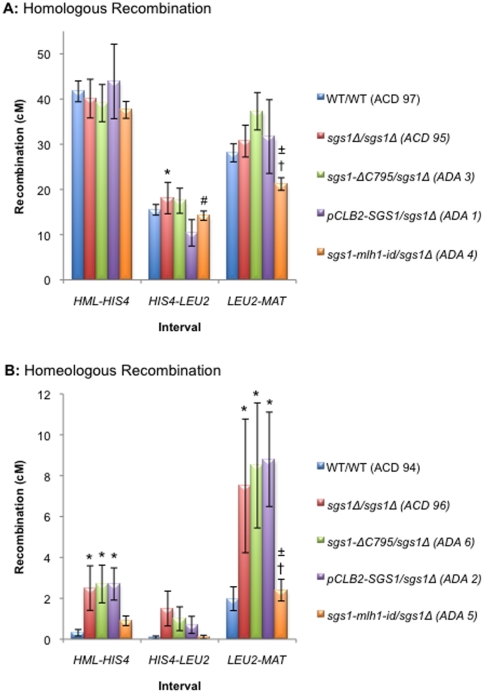
Map distances on chromosome III. (**A**) Homologous recombination. **B**) Homeologous recombination. Map distances were calculated using the Perkins formula [85]. The distribution of PDs, NPDs and TTs for homologous diploids was compared using the G-test. After correcting for multiple comparisons using the Benjamini-Hochberg correction [86], p-values <0.05 were considered significant. The standard error of the map distances was calculated using Stahl Online Tools (http://molbio.uoregon.edu/~fstahl/compare2.php). * - significantly different from WT/WT; # - significantly different from *sgs1Δ*/*sgs1Δ*; † - significantly different from *sgs1-ΔC795*/*sgs1Δ*; ± - significantly different from *pCLB2-SGS1*/*sgs1Δ*.

**Figure 4:**
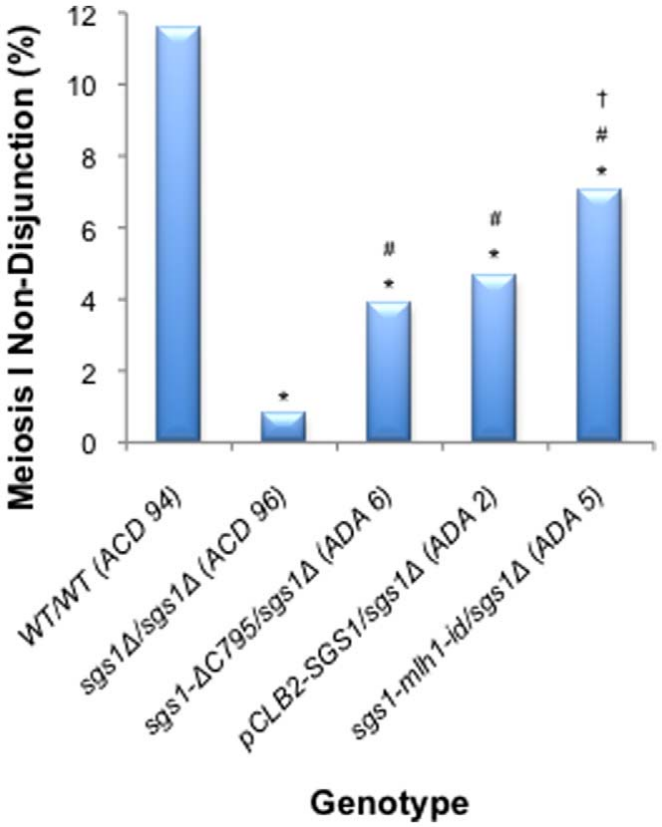
Frequency of meiosis I non-disjunction events for homeologous diploids. Meiosis I non-disjunction events on chromosome III were identified in homeologous diploids as described in *Methods and Materials*. Frequencies of meiosis I non-disjunction events out of the total number of tetrads dissected were compared using the G-test. After correcting for multiple comparisons using the Benjamini-Hochberg correction [86], p-values <0.05 were considered significant. * - significantly different from WT/WT; # - significantly different from *sgs1Δ/sgs1Δ*.

To investigate the involvement of Sgs1 in the suppression of homeologous recombination, we assessed the levels of crossing over in three genetic intervals along chromosome III: *HML:ADE1-HIS4*, *HIS4-LEU2* and *LEU2-MAT* (**Figure 3** and **Tables S3** and **S4**) in various mutant strains. In addition we assessed the levels of meiosis I non-disjunction for the partial hybrid diploids. As shown in **Figure 1**, failure to cross over in a single chromosome pair leads to meiosis I non-disjunction and inviability of the two nullisomic spores. The two remaining viable spores are disomic, and because the mating-type cassettes are located on chromosome III, these will be non-maters. We measured the proportion of the two viable spore class of tetrads that were caused by non-disjunction of the homeologous chromosome IIIs by checking the mating status of the viable spore colonies (**Figure 4** and **Table S5**).

As seen previously, the presence of sequence divergence significantly decreases the levels of wild-type recombination (*ACD 94*) 132-fold, 196-fold and 14-fold in *HML-HIS4*, *HIS4-LEU2* and *LEU2-MAT* respectively, when compared to the homologous cross (*ACD 97*) (**Figure 3** and **Tables S3** and **S4**) [24]. The greatest decrease is seen in the *HIS4-LEU2* interval. Consistent with the decrease in crossing over observed for the homeologous cross (**Figure 3B**), we saw a high level of meiosis I non-disjunction (11.5%) similar to that seen previously for the wild type hybrid diploid [24] (**Figure 4** and **Table S5** – *ACD 94*).

Previously, it has been shown that a deletion of *SGS1* causes a modest yet significant increase in homologous recombination [10], [54], [56]. We failed to reproduce these observations in two of the three intervals studied (**Figure 3A** – *ACD 95*). This may be due to the different strain backgrounds and sporulation conditions used in these studies [70]. However, when sequence divergence was present, we observed a significant increase in recombination for *sgs1Δ*/*sgs1Δ* (*ACD 96*), *sgs1-ΔC795/sgs1Δ* (*ADA 6*) and *pCLB2-SGS1*/*sgs1Δ* (ADA 2) in two of the three intervals analysed. Furthermore, we also observed a significant decrease in the levels of meiosis I non-disjunction when *SGS1* is deleted in the partial hybrid (4.6% for *pCLB2-SGS1/sgs1Δ*; p = 2.11×10^-6^ – *ADA 2*) (**Figure 4** and **Table S5**). These data suggest that the absence of Sgs1 facilitates recombination between diverged sequences and, in doing so, decreases the likelihood of chromosome mis-segregation.

The failure to observe a significant effect on crossing over in the *HIS4-LEU2* interval between the homeologous chromosomes might be due to fewer successful strand invasions as a consequence of a higher degree of sequence divergence present in this interval. In order to determine whether the variation in the map distances amongst the three intervals was related to different levels of sequence divergence we calculated the sequence identity for each interval as described in the *Methods and Materials*. The sequence identity for *HML-HIS4*, *HIS4-LEU2* and *LEU2-MAT* was 88.4%, 85.6% and 88.5%, respectively. While the degree of sequence divergence between the intervals appears to be small, it still may be sufficient to account for the variation in fold reduction in crossing over, as large effects due to small changes in divergence have been noted before [60]. Additionally, despite not seeing any significant difference in the *HIS4-LEU2* interval, the data shown in **Figure 3B** and **Table S4** suggests that deletion of *SGS1* in the *HIS4-LEU2* interval does cause an increase in map distance. Thus, a second possible explanation as to why we do not observe any significant difference may be due to the number of tetrads analysed in this study. In order to increase the size of the data set, we tested the data obtained for the different mutations of *SGS1* (*sgs1Δ/sgs1Δ*, *sgs1-ΔC795/sgs1Δ* and *pCLB2-SGS1/sgs1Δ*) for homogeneity by comparing the distribution of PDs, NPDs and TTs using the G-test. The data from these crosses were not significantly different from each other for either the homologous or homeologous diploids. This allowed us to pool the data for all three *SGS1* mutant strains and to reanalyse the effect of deleting *SGS1* on homeologous recombination collectively (**Table S4**). We observed a significant increase in recombination for the combined *SGS1* mutant data when compared to wild type in all three intervals. This suggests that crossovers between diverged sequences are rescued in the absence of *SGS1* in the *HIS4-LEU2* interval as well as the other two intervals.

Sgs1 is known to interact with the MMR protein Mlh1 [63], [64]. We hypothesised that Mlh1 might act as a ‘molecular matchmaker’ between the MMR complex and Sgs1 to facilitate the unwinding of homeologous recombination intermediates. To test this, we analysed the effects of a mutation that disrupts the ability of Sgs1 to interact with Mlh1 (*sgs1-mlh1-id*) [64]. We saw a significant decrease in the levels of meiosis I non-disjunction for *sgs1-mlh1-id*/*sgs1*Δ (*ADA 5*; p = 0.0003) when compared to wild type, equivalent to that seen for *pCLB2-SGS1*/*sgs1*Δ (*ADA 2*) (**Figure 4**). These data indirectly suggest that the interaction between Sgs1 and Mlh1 is important for the ability of Sgs1 to suppress meiotic homeologous recombination. However, there is no obvious effect on crossing over between the homeologous chromosomes in the *sgs1-mlh1-id*/*sgs1*Δ (*ADA 5*) strain (**Figure 3B**).

### Does Sgs1 aid in the completion of reciprocal homeologous recombination?

As described in the *Introduction*, Chambers *et al*. [24] proposed that the MMR proteins Msh2 and Pms1 act to suppress meiotic homeologous recombination at the strand capture stage. They based this proposal on the observation that recombination was six-fold higher in tetrads with only three viable spores than in the four viable spore tetrads and that the dead spore was preferentially recombinant. This phenotype was abolished in MMR defective strains. To assess the potential role of Sgs1 in this phenomenon, we determined if the increase in the rate of recombination for the three viable spore class of tetrads compared to the four viable spore class of tetrads was dependent on *SGS1* (**Table 1**). As seen previously [24], there was a significant increase in recombination for the three viable spore class of tetrads in the wild type (*ACD 94*). However, when Sgs1 was repressed during meiosis (*pCLB2-SGS1/sgs1*Δ – *ADA 2*), this increase was no longer seen, suggesting that Sgs1 aids in rejecting strand capture of the reciprocal product in the presence of mismatches. This activity does not seem to be dependent on the interaction between Sgs1 and Mlh1 as crossing over was still significantly enriched in the three viable spore class of tetrads from the *sgs1-mlh1-id*/*sgs1*Δ cross (*ADA 5*) (**Table 1**). This is consistent with the observation that abolishing the interaction with Mlh1 did not have any effect on increasing recombination in the four viable spore class of tetrads discussed above.

**Table 1:** Map distances in the four and three viable spore classes of tetrads in homeologous diploids.

Homeologous Diploid	Interval	Tetrad Class	PD	NPD	TT	Recombination (cM)	p-value
WT/WT (ACD 94)	*HML-HIS4*	4 Viable	626	0	4	0.317	2.87×10^−6^ *
		3 Viable	147	0	13	4.06	
	*HIS4-LEU2*	4 Viable	630	0	1	0.079	0.218
		3 Viable	158	0	2	0.625	
	LEU2-MAT	4 Viable	611	1	19	1.981	4.97×10^−8^ *
		3 Viable	126	2	24	11.84	
pCLB2-SGS1/sgs1Δ (ADA 2)	*HML-HIS4*	4 Viable	194	0	11	2.7	0.736
		3 Viable	151	0	12	3.7	
	*HIS4-LEU2*	4 Viable	202	0	3	0.7	0.25
		3 Viable	156	0	7	2.1	
	LEU2-MAT	4 Viable	179	2	24	8.8	0.86
		3 Viable	140	2	22	10.4	
sgs1-mlh1-id/sgs1Δ (ADA 5)	*HML-HIS4*	4 Viable	744	0	13	0.9	0.0002 *
		3 Viable	143	0	14	4.5	
	*HIS4-LEU2*	4 Viable	756	0	2	0.1	0.0065 *
		3 Viable	153	0	5	1.6	
	LEU2-MAT	4 Viable	728	1	31	2.4	4.27×10^−8^ *
		3 Viable	129	0	29	9.2	

The distribution of PDs, NPDs and TTs for the three viable spore class of tetrads were compared to the four viable spore class of tetrads. p-values <0.05 were considered significant using the G-test (denoted by *).

There are a number if possible explanations for why we do not observe a significant improvement in crossing over for the *sgs1-mlh1-id* mutant. One possibility is that because Sgs1 also interacts with Mlh3 [71] and Msh6 [72], these interactions might be sufficient to facilitate the formation of a stable complex in order to carry out anti-recombination either by heteroduplex rejection (Msh6) or dissolving (Msh3). Another possibility is that tetrad analysis only measures crossovers across three intervals comprising approximately only 61% of the chromosome, while the meiosis I non-disjunction gives an indication of a failure of crossing over across the entire length of chromosome III. This would suggest that the levels of meiosis I non-disjunction are a more accurate reflection of the need for Sgs1 to interact with Mlh1 in the suppression of meiotic homeologous recombination. Another possibility is that since Sgs1 needs to interact with Mlh1 to unwind/dissolve sister chromatid events (*see below*) it may be that the existence of these inter-sister events somehow aids segregation in the homologue. Finally, we cannot exclude the possibility that Sgs1 plays a role in suppressing the segregation of non-exchange chromosomes and that this activity is dependent on an interaction with Mlh1.

### Sgs1 suppresses sister chromatid exchange

**Figure 5:**
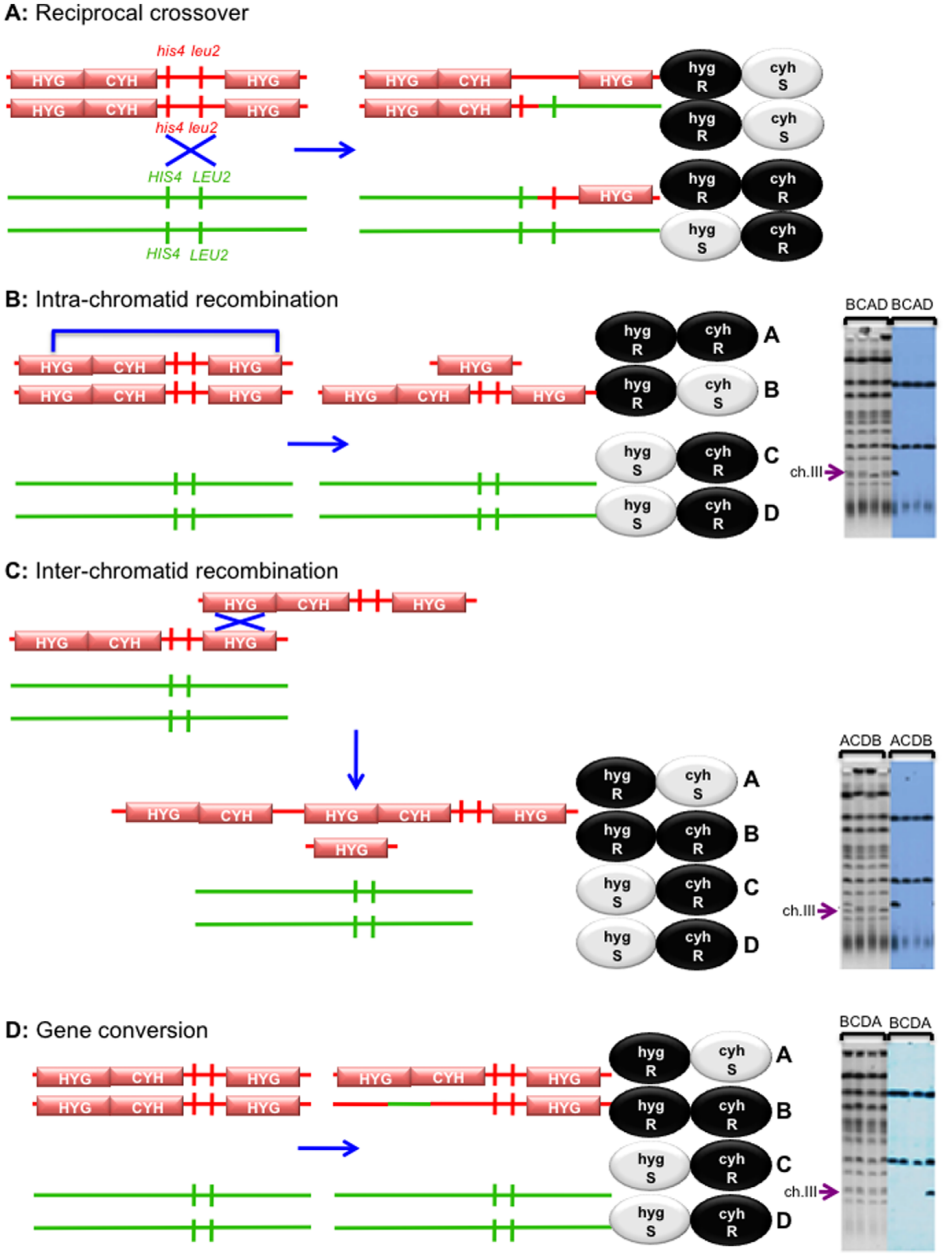
Unequal Recombination. A strain containing the HYG-CYH/HYG cassette (in red) was mated to a strain that does not (in green) to assess unequal recombination events. The genetic (drug resistance/sensitivity) phenotype and physical karyotype of all four spores from each type of recombination event are illustrated. **A:** A **reciprocal** **crossover** event that occurs between *HIS4* and *LEU2*, leading to 3 Hyg^R^: 1 Hyg^S^ and 2 Cyh^S^: 2 Cyh^R^. **B: Intra-chromatid** events (or Deletion events). Crossing over between the hygromycin cassettes on the same sister strand lead to a deletion event. This is seen as 2 Hyg^R^: 2 Hyg^S^ and 1 Cyh^S^: 3 Cyh^R^ segregation patterns. Four lanes of a CHEF Gel are shown, each of which represents one spore of a four viable spore tetrad. Chromosome III is indicated with an arrow (**→**). Due to the deletion event, approximately 27.5kb DNA will have been lost. This results in the absence of a band where expected and a band of double intensity below, as chromosome III now migrates with chromosome VI. When probed with *URA3* and *CYH2* sequences, the *URA3* containing chromosome V (top band) and the *CYH2* containing chromosome VII (middle band) are labelled. Chromosome III (bottom band) is labelled when it retains the *CYH2* insert. Thus, the smaller chromosome III, which has deleted all of the sequences between *HIS4* and *LEU2*, is unlabelled. **C:** **Inter-chromatid** events (or Unequal Sister Chromatid Exchange events). When a reciprocal crossover occurs between one hygromycin cassette on one sister strand and the other hygromycin cassette on the second sister strand, a triplication event and a deletion event are seen as 2 Hyg^R^: 2 Hyg^S^ and 1 Cyh^S^: 3 Cyh^R^ colonies. The triplication event results in chromosome III migrating more slowly, while the deletion event migrates faster (as discussed in **B**). Southern blot analysis is used as physical confirmation of the genetic diagnosis, as discussed above (**B**). **D:** **Gene Conversion** events. Tetrads that are 2 Hyg^R^: 2 Hyg^S^ and 1 Cyh^S^: 3 Cyh^R^ can also arise by gene conversion of the *HYG-CYH* region. Because a gene conversion event does not result in a major size change, the CHEF karyotype is normal. However, Southern blotting indicates that one copy of the *CYH2* gene has been replaced with wild-type sequences.

Physical studies have implicated Sgs1 in suppressing inter-sister joint molecules [54], [55], [57], [58]. In order to genetically investigate this role in meiosis, we designed a reporter construct on chromosome III that detects unequal intra-chromosomal recombination events (described in *Methods and Materials* and **Figure 5**). The frequencies and distributions of unequal recombination events are given in **Tables 2** and **3**. The frequency of gene conversion of the *HYG-CYH2* insert in the *SGS1* mutations does not significantly differ from wild type (*ACD 97*) (**Table 2**). However, mutation of *SGS1* (*ACD 116*) leads to an increase in the rates of unequal recombination when compared to wild type (**Table 3**), as evaluated using the G-test for homogeneity (p = 0.0005). This is in agreement with previous genetic studies that showed an elevation in sister-chromatid recombination during mitosis [29], and with physical studies showing increases in inter-sister joint molecules during meiosis [55], [57] when *SGS1* is deleted. The *sgs1-top3-id*/*sgs1*Δ cross (*ADA 12*) and the *sgs1-mlh1-id*/*sgs1*Δ cross (*ADA 4*) also show a significant increase in the number of unequal recombination events (both USCE and deletions) compared to wild type (p = 0.0267 and p = 0.0079, respectively). The data therefore suggest that the suppression of unequal recombination by Sgs1 is dependent on its interactions with Top3, presumably by recruiting Top3 to dissolve dHJs. Previous studies have suggested that Msh4, Msh5, Mlh3 (and Mlh1 by inference) act to protect inter-homolog recombination intermediates from dissolution by Sgs1 [54], [55]. In addition, Mlh3 (and presumably Mlh1), Sgs1 and Top3 have been shown to form a complex during meiosis [71] which is inconsistent with the sole function of Mlh1 and Mlh3 being to block dissolution. Our observations suggest that the interaction of Sgs1 with Mlh1 is important for Sgs1 to carry out its anti-recombination activity, at least as it relates to preventing inter-sister recombination. We propose that the role of Mlh1 may be to recruit Sgs1 to unwind/dissolve inappropriate inter-sister recombination events [71], perhaps mediated by structures recognized by Msh2/Msh3 [73], [74]. Thus, in the absence of interactions between Sgs1 and Mlh1/Top3, Top3-mediated dissolution of these unequal recombination intermediates cannot be carried out.

**Table 2:** Gene conversion events for homologous diploids.

Homologous Diploid	Number of gene conversion events	Tetrads that did not exhibit a gene conversion	Total Number of tetrads	Percentage of gene conversion events
WT/WT (ACD 97)	11	244	255	4.3
sgs1Δ/sgs1Δ (ACD 116)	5	173	178	2.8
sgs1-ΔC795/sgs1Δ (ADA 3)	4	173	177	2.6
sgs1-mlh1-id/sgs1Δ (ADA 4)	8	413	421	1.9
sgs1-top3-id/sgs1Δ (ADA 12)	4	221	225	1.8

**Table 3:** Unequal recombination events for homologous diploids.

	Unequal Recombination Events				
Homologous Diploid	USCE Events	Deletion Events	Tetrads that did not exhibit an unequal recombination event	Total Number of tetrads	Percentage of unequal recombination events
WT/WT (ACD 97)	6	0	249	255	2.4
sgs1Δ/sgs1Δ (ACD 116)	13	5	160	178	10.1 *
sgs1-ΔC795/sgs1Δ (ADA 3)	4	8	165	177	6.8 *
sgs1-mlh1-id/sgs1Δ (ADA 4)	8	10	403	421	4.3 *#
sgs1-top3-id/sgs1Δ(ADA 12)	9	4	212	225	5.78 *

The distribution of classes of events amongst wild type and *SGS1* mutant strains were compared using the G-test. p-values <0.05 were considered significant.

* = significantly different from WT; # = significantly different from *sgs1Δ*.

### Deletion of *SGS1* leads to an increase in the number of non-sister spores in the two viable spore class of tetrads

**Figure 6:**
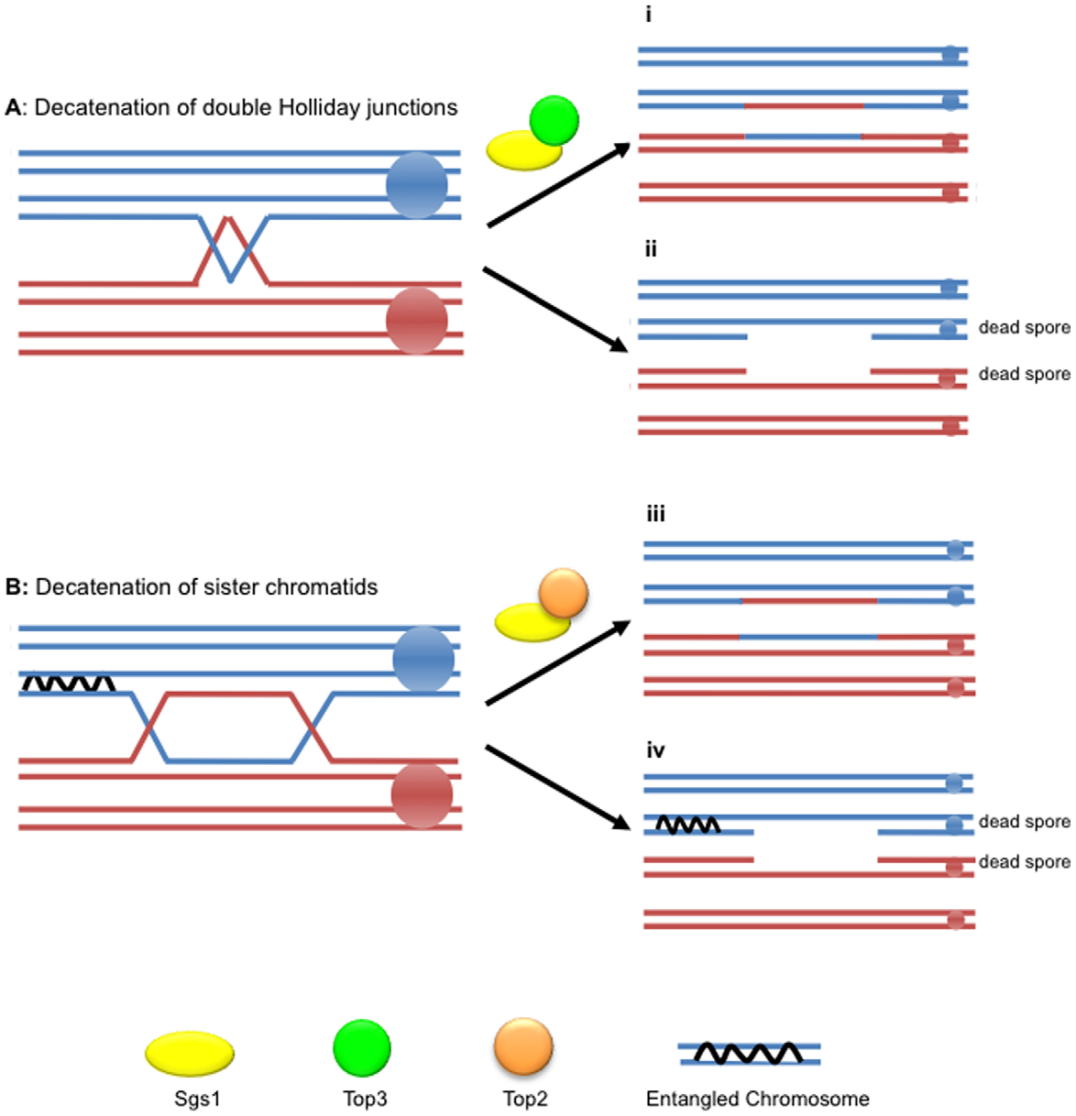
Failure to decatenate sister chromatids can lead to spore inviability. **A**: Sgs1 acts in the decatenation of dHJ structures with Top3 late during meiosis. Sgs1 and Top3 act to dissolve double Holliday junctions (**i**). In the absence of this interaction, the double Holliday junction is not dissolved leading to destruction of the entangled chromosomes and inviability of the non-sister spores (**ii**). **B**: Sgs1 interacts with Top2 to decatenate sister chromatids arising from pre-meiotic replication. Failure to decatenate sister chromatids (**iii**) lead to the inability of recombined chromosomes to segregate. The entanglement can lead to chromosome breakage and/or chromosome loss (**iv**). Because the crossover links the non-sister centromeres, the two remaining viable spores are also non-sisters.

Using the centromere marker *TRP1*, we were able to assess the frequency of sister and non-sister spores in the two viable spore class of tetrads (**Figure 1C** and **Table 4**). Sister spores contain the same centromere allele and therefore both will either be prototrophic or auxotrophic for growth on tryptophan. Both classes arise with equal frequencies if spore death is due to random causes. However, a significant bias toward the recovery of sister spores is a hallmark of meiosis I non-disjunction. When analysing spore viability in homologous *SGS1* mutant strains, we noted a significant increase in the number of two viable spore tetrads for *sgs1*Δ/*sgs1*Δ (27.08%) and for *pCLB2-SGS1*/*sgs1*Δ (19.1%) compared to wild type (0.9%). These could not be attributed to meiosis I non-disjunction as they were significantly enriched for non-sister spores (**Figure 1C**). An increase in non-sister spores has not been reported previously but could be predicted based on the known activities of Sgs1 (**Figure 6**). As shown in **Figure 6A**, Sgs1 and Top3 are proposed to act in the dissolution of dHJ structures [37]–[41]. When *SGS1* is deleted, these structures cannot be dissolved. Thus, one possibility is that the failure to dissolve the interacting homologues leads to breakage of the entangled chromosomes and death of the spores containing them [57]. The two surviving spores will be non-sisters. Alternatively, Sgs1 is known to interact with Top2 [50], [51] and this interaction may be required for decatenating sister chromatids after pre-meiotic replication (**Figure 6B**). If this is the case, when there is a crossover between the entangled region and the centromere, failure to decatenate would lead to broken chromosomes and the death of two spores. The two remaining spores would be non-sisters.

**Table 4:** Distribution of sister and non-sister spores.

	2 viable spore class of tetrads		
Homologous Diploids	Sisters	Non-Sisters	p-value
sgs1Δ/sgs1Δ (ACD 95)	110 (36.54%)	191 (63.46%)	3×10^−6^ *
sgs1-ΔC795/sgs1Δ (ADA 3)	56 (38.62%)	89 (61.38%)	0.006 *
pCLB2-SGS1/sgs1Δ (ADA 1)	3 (14.29%)	18 (85.71%)	0.001 *
sgs1-top3-id/sgs1Δ (ADA 12)	47 (43.52%)	61 (56.48%)	0.178

p-values <0.05 were considered significant using χ^2^ test (significance denoted by *) which indicated that the ratio of sister : non-sister spores deviated significantly from 50∶50.

To test the model shown in **Figure 6A**, we assessed whether the *sgs1-top3-id* mutation also results in an elevation of non-sister spores. There is no significant difference between the frequency of sister and non-sister spores in the *sgs1-top3-id/sgs1*Δ cross (*ADA 12*) when compared to wild type (**Table 4**). This suggests that the preferential death of non-sister spores in the absence of Sgs1 does not result from a Top3-dependent inability to dissolve dHJs. Support for this idea comes from the analysis of the *sgs1-*Δ*C795*/*sgs1*Δ cross (*ADA 3*) which also shows an increase in the number of non-sister spores compared to sister spores (**Table 4**) despite Sgs1 retaining the ability to interact with Top3 [75]. However, the Top2-interacting domain of Sgs1 has been disrupted in this mutant [50], [51]. Therefore, it is possible that the inability of Sgs1 to interact with Top2 in both the *sgs1*Δ/*sgs1*Δ and *sgs1-*Δ*C795*/*sgs1*Δ mutants leads to the increase in non-sister spores due to the failure to decatenate sister chromatids after replication, as suggested in **Figure 6B**.

### Summary

The data presented in this study highlight the importance of Sgs1 in the early stages of meiosis. Firstly, based on the discovery of an unusual type of tetrad class, we propose a role for Sgs1 and Top2 in the decatenation of entangled chromosomes prior to entry into meiosis. Secondly, we propose that Sgs1 unwinds recombination events between homeologous chromosomes at the strand invasion stage and the strand capture stage. Finally, the data presented here are most consistent with the role of Sgs1 in the prevention of unequal sister chromatid exchange being carried out via Top3-mediated dissolution aided, in an unknown fashion, by an interaction between Sgs1 and Mlh1.

## Materials and Methods

### Strains

All of the strains used in this study are in a Y55 background and are listed in **Table S1**. Diploid strains are listed in **Table S2**. Deletion strains were made by deleting the coding region of the relevant gene with a KANMX4 cassette [76]. *sgs1-ΔC795* [75] was made by replacing the sequences downstream from amino acid 652 with a NATMX4 cassette [77]. As an *sgs1* null leads to defects during both mitosis and meiosis, in addition to analysing a complete deletion of *SGS1* (ACT 2), we also investigated the effects of eliminating meiotic transcription of *SGS1* by replacing the native promoter of *SGS1* with the promoter of the *CLB2* gene (Y55 3565) [54], [57], [58], [69]. *sgs1-top3-id* was made using site directed mutagenesis (using the *pJET* cloning kit by *Ferme*ntas) to change the amino acids at positions 4, 5 and 9 into alanine residues [49]. *sgs1-mlh1-id* was made by changing the amino acids at positions 1383, 1385 and 1386 to alanine residues [64]. Mutations were introduced into yeast by transformation [78] followed by selection on 5-FOA [79]. The oligonucleotides used for the construction of these strains are listed in **Table S6**.

In order to determine whether the mutant alleles made for this study (**Figure 2**) were functional in mitosis, we assayed growth on YEPD plates supplemented with 0.02% methyl methanesulfonate (MMS) (**Figure S1**). As previously shown, deletion of *SGS1* resulted in sensitivity to MMS [28], [49], [75]. In both the homologous (Y55 3567) and homeologous (Y55 3565) strains expressing *pCLB2-SGS1*, resistance to MMS was normal. Thus, *pCLB2-SGS1* expresses sufficient amounts of the Sgs1 protein to fulfil its mitotic roles. Jessop *et al*. [54] have demonstrated that expression of the Sgs1 protein in a *pCLB2-SGS1* strain is repressed approximately 2 hours after the onset of sporulation, with no noticeable traces of the protein after 4 hours. Other groups have also confirmed genetically that *pCLB2-SGS1* is a meiotic null [57]. Several groups have shown that deletion or mutation of part of the N-terminus of Sgs1, responsible for binding Top3, renders the strains sensitive to MMS [28], [49], [75], [80], [81]. In agreement with this, we saw that *sgs1-K4A,P5A,L9A*, which fails to bind Top3 [49], leads to sensitivity to MMS.

### Sequence Alignment Between *S. cerevisiae* chromosome III and *S. paradoxus* chromosome III

The sequences for the *HML-HIS4*, *HIS4-LEU2* and *LEU2-MAT* intervals were downloaded from the *Saccharomyces Genome Database* (http://yeastgenome.org/). The sequence for chromosome III of *S. paradoxus* was downloaded from the Wellcome Trust Sanger Institute's *Saccharomyces Genome Resequencing* webpage (http://www.sanger.ac.uk/research/projects/genomeinformatics/sgrp.html). For the *HML-HIS4* interval, we used the *S. cerevisiae* sequences outside of *HML* to the nucleotide before the stop codon of *HIS4* (chromosomal co-ordinates 14850–65933). For the *HIS4-LEU2* interval, we used the *S. cerevisiae* sequences that start at the *HIS4* stop codon and end at the stop codon of *LEU2* (chromosomal co-ordinates 65934–92418). For the *LEU2-MAT* interval, we used the *S. cerevisiae* sequences from the nucleotide after the start codon of *LEU2* to the nucleotide before the start codon of MAT-alpha (chromosomal co-ordinates 92419–198667). This allowed us to align non-overlapping intervals of *S. cerevisiae* to chromosome III of *S. paradoxus*. The sequences were aligned using the *NUCmer* alignment software (Ver. 3.06), part of the open source mummer suite [82] (http://mummer.sourceforge.net/), to determine the degree of sequence identity for each interval. Each interval was aligned to the *S. paradoxus* chromosome III using the NUCmer default parameters and the –coords option to generate a table of aligned sections.

### Tetrad Dissection and Analysis

Diploids were sporulated for 3–5 days at 23°C on complete 2% potassium acetate solid medium (as described previously [83], [84]) and asci were separated by micromanipulation using a Zeiss dissecting microscope. After dissection, plates were replicated on various synthetic media in order to study the segregation of markers as described previously [84]. Map distances were calculated using the Perkins formula [85]. To analyse three viable spore tetrads, the genotype of the dead spore was predicated by analysing the genotypes of the viable spores assuming Mendelian segregation. The distribution of classes of tetrads were analysed using the G-test. As multiple comparisons were made, the Benjamini-Hochberg correction was applied [86] to limit the false discovery rate (http://udel.edu/~mcdonald/statmultcomp.html).

Sister and non-sister spores were classified by the pattern of the centromere marker *TRP1*. In the two viable spore class of tetrads, sisters were identified if both viable spores were auxotrophic or prototrophic for tryptophan. Non-sister spores were identified if one spore was auxotrophic and the other was prototrophic for tryptophan.

Mating phenotype was determined by crossing with appropriate tester strains. Meiosis I non-disjunction (**Figure 1B**) leads to two copies of chromosome III from each parent. This means that the spores will contain both MATa and MATα information and will therefore be non-maters.

### Unequal Recombination Assay

One copy of a hygromycin resistance cassette [77] and one copy of the *CYH2* gene were inserted upstream of *HIS4* (at chromosomal co-ordinate 65822) and a single copy of the hygromycin resistance cassette was inserted downstream of *LEU2* on chromosome III (at chromosomal co-ordinates 92654 – 92667). *CYH2* is dominant to the *cyh2-r* allele on chromosome VII. The rates of unequal recombination can be measured using the segregation of the Hyg^R^ and Cyh^R^ phenotypes. As shown in **Figure 5**, unequal recombination (caused by either a deletion (**Figure 5B**) or by unequal sister chromatid exchange (**Figure 5C**)) can be determined by assessing the number of four viable spore tetrads exhibiting 3 Hyg^R^: 1 Hyg^S^ and 2 Cyh^S^: 2 Cyh^R^. However, 3 Hyg^R^: 1 Hyg^S^ and 2 Cyh^S^: 2 Cyh^R^ can also be caused by gene conversion (**Figure 5D**). As described below (in the *Methods and Materials* section), CHEF Gel and Southern Blot analysis allows us to differentiate between these events (**Figure 5**). Deletions between the hygromycin cassettes can be recovered, as there are no essential genes between the inserts.

### Contour-Clamped Homogeneous Electric Field (CHEF) Gels to separate *S. cerevisiae* chromosomes

The CHEF DRIII system (Bio-Rad) was used to separate the chromosomes of *S. cerevisiae*. DNA was prepared for CHEF Gel analysis as described by Louis and Haber [87]. To obtain good separation of the smallest chromosomes gels were run for 15 hours with a 60 second switching time followed by 9 hours with a 90 second switching time. Gels were run at 14°C in 0.5× TBE (0.045M Tris-borate, 0.045M boric acid and 0.001M EDTA) at 6 volts/cm and at an angle of 120°.

### Southern Blot Analysis

Southern blotting was carried out as described in Sambrook *et al*. [88]. The DNA probe was prepared using the DIG-High Prime system (Roche) as described in the manufacturer's instructions.

## Supporting Information

Table S1 Haploid strain list. (DOC)

Table S2 Diploid strain list. (DOC)

Table S3 Map distance for intervals along chromosome III for homologous diploids. The distribution of PDs, NPDs and TTs for homologous diploids were compared using the G-test. After correcting for multiple comparisons using the Benjamini-Hochberg correction [86], p-values <0.05 were considered significant. The map distances for both the homologous diploids are shown in **Figure 4A**. * - significantly different from WT/WT; # - significantly different from *sgs1Δ*/*sgs1Δ*; † - significantly different from *sgs1-ΔC795*/*sgs1Δ*; ± - significantly different from *pCLB2-SGS1*/*sgs1Δ*; § = significantly different from *sgs1Δ* combined. (DOC)

Table S4 Map distance for intervals along chromosome III for homeologous diploids. The distribution of PDs, NPDs and TTs for homeologous diploids were compared using the G-test. After correcting for multiple comparisons using the Benjamini-Hochberg correction [86], p-values <0.05 were considered significant. The map distances for both the homeologous diploids are shown in **Figure 4B**. ^a^ – *sgs1Δ* combined represents the collective data from the *sgs1Δ/sgs1Δ* (ACD 96), *sgs1-ΔC795/sgs1Δ* (ADA 6) and *pCLB2-SGS1/sgs1Δ* (ADA 2) homeologous crosses. * - significantly different from WT/WT; # - significantly different from *sgs1Δ*/*sgs1Δ*; † - significantly different from *sgs1-ΔC795*/*sgs1Δ*; ± - significantly different from *pCLB2-SGS1*/*sgs1Δ*; § = significantly different from *sgs1Δ* combined. (DOC)

Table S5 Meiosis I non-disjunction events in homeologous diploids. Meiosis I non-disjunction events on chromosome III were identified in homeologous diploids as described in *Methods and Materials*. Frequencies of meiosis I non-disjunction events out of the total number of tetrads dissected were compared using the G-test. After correcting for multiple comparisons using the Benjamini-Hochberg correction [86], p-values <0.05 were considered significant. * - significantly different from WT/WT; # - significantly different from *sgs1Δ/sgs1Δ*; † - significantly different from *sgs1-ΔC795*/*sgs1Δ*. (DOC)

Table S6 Oligonucleotides used in this study. Bold sequences, as described by Longtine *et al* [89], are homologous to *pA6a-KANMX6-pCLB2-3HA* plasmid [69]. Underlined sequences are homologous to the *pAG25* (*NATMX4*) and *pAG32* (*HYGMX4*) plasmid [77]. (DOC)

Figure S1 Testing the growth of different *sgs1* mutants with respect to MMS resistance by spotting serial dilutions onto YEPD plates (as a control) and YEPD plates supplemented with 0.02% MMS. Failure to grow on YEPD media supplemented with 0.02% MMS is indicative of an inability to repair lesions which lead to the stalling of replication forks during mitosis. (TIFF)
