# Supplementary material for: The Roles of the Saccharomyces cerevisiae RecQ Helicase SGS1 in Meiotic Genome Surveillance
Source: PLoS One. 2010 Nov 9;5(11):e15380. doi: 10.1371/journal.pone.0015380 (PMC2976770; doi:10.1371/journal.pone.0015380)
Supplement: Table S1 — Haploid strain list. (DOC) [file pone.0015380.s001.doc]

**Table S1:** Haploid strain list*

| **Strains** | **Key Features** | **Genotype** |
| --- | --- | --- |
| ACT 2 | *S. paradoxus* N17 *sgs1∆* | *ade1-1; α; ho∆; met13-4; ura3::nco; cyh2-1; kar1∆13; sgs1::KANMX4* |
| ACT 53 | Wild type | *ade1-1; α; ho∆; met13-2; ura3::nco; cyh2-1* |
| ACT 56 | *sgs1∆* | *ade1-1; α; hoΔ; met13-2; ura3::nco; cyh2-1; sgs1::KANX4* |
| ACT 57 | Wild Type | *ade1-1; HML::ADE1; his4-r1; leu2-r1; α; ho∆; lys2-c; met13-2; ura3::nco; trp1:bsu36; cyh2-1* |
| ACT 65 | *HYG-CYH/HYG* | *ade1-1; HML::ADE1; HYG-CYH2-his4-r1; leu2-r1-HYG; a; ho∆; lys2-c; met13-2; ura3::nco; trp1::bsu36; cyh2-1* |
| ACT 66 | *HYG-CYH/HYG sgs1∆* | *ade1-1; HML::ADE1; HYG-CYH2-his4-r1; leu2-r1-HYG; a; ho∆; lys2-c; met13-2; ura3::nco; trp1::bsu36; cyh2-1; sgs1::KANMX4* |
| ACT 83-1 | *sgs1-∆C795* | *ade1-1; α; ho∆; met13-2; ura3::nco; cyh2-1; sgs1∆-C795::NATMX4* |
| Y55 3544 | *sgs1-mlh1-id* | *ade1-1; HML::ADE1; HYG-CYH2-his4-r1; leu2-r1-HYG; a; hoΔ; lys2-c; met13-2; ura3::nco; trp1::bsu36; cyh2-1; sgs1-S1383A,F1385A,F1386A* |
| Y55 3541 | *sgs1-top3-id* | *ade1-1; HML::ADE1; HYG-CYH2-his4-r1; leu2-r1-HYG; a; hoΔ; lys2-c; met13-2; ura3::nco; trp1::bsu36; cyh2-1; sgs1-K4A,P5A,L9A* |
| Y55 3565 | *S. paradoxus* N17 *pCLB2-SGS1* | *ade1-1; α; ho∆; met13-4; ura3::nco; cyh2-1; kar1∆13; KANMX6::pCLB2-SGS1* |
| Y55 3567 | *pCLB2-SGS1* | *ade1-1; α; ho∆; met13-2; ura3::nco; cyh2-1; KANMX6::pCLB2-SGS1* |

*All strains used in this study are in a Y55 background
