# Supplementary material for: The Roles of the Saccharomyces cerevisiae RecQ Helicase SGS1 in Meiotic Genome Surveillance
Source: PLoS One. 2010 Nov 9;5(11):e15380. doi: 10.1371/journal.pone.0015380 (PMC2976770; doi:10.1371/journal.pone.0015380)
Supplement: Table S2 — Diploid strain list. (DOC) [file pone.0015380.s002.doc]

**Table S2:** Diploid strain list

| **Diploid Strains** | **Haploid Strains** | **Genotype** | **Key Features** |
| --- | --- | --- | --- |
| ACD 94 | SCT 14 | *ade1-1 LYS2 HML HIS4 LEU2 α hoΔ TRP1 ura3::nco met13-4 cyh2-1 kar1∆13* | WT/WT homeologous |
|  | ACT 65 | *ade1-1 lys2-c HML::ADE1 HYG-CYH2-his4-r1 leu2-r1-HYG a hoΔ trp1::bsu36 ura3::nco met13-2 cyh2-1 KAR1* |  |
| ACD 95 | ACT 56 | *ade1-1 LYS2 HML HIS4 LEU2 α hoΔ TRP1 ura3::nco met13-2 cyh2-1 sgs1::KANMX4* | *sgs1Δ*/*sgs1Δ* homologous |
|  | ACT 66 | *ade1-1 lys2-c HML::ADE1 HYG-CYH2-his4-r1 leu2-r1-HYG a hoΔ trp1::bsu36 ura3::nco met13-2 cyh2-1 sgs1::KANMX4* |  |
| ACD 96 | ACT 2 | *ade1-1 LYS2 HML HIS4 LEU2 α hoΔ TRP1 ura3::nco met13-4 cyh2-1 sgs1::KANMX4 kar1∆13* | *sgs1Δ*/*sgs1Δ* homeologous |
|  | ACT 66 | *ade1-1 lys2-c HML::ADE1 HYG-CYH2-his4-r1 leu2-r1-HYG a hoΔ trp1::bsu36 ura3::nco met13-2 cyh2-1 sgs1::KANMX4 KAR1* |  |
| ACD 97 | ACT 53 | *ade1-1 LYS2 HML HIS4 LEU2 α hoΔ TRP1 ura3::nco met13-2 cyh2-1* | WT/WT homologous |
|  | ACT 65 | *ade1-1 lys2-c HML::ADE1 HYG-CYH2-his4-r1 leu2-r1-HYG a hoΔ trp1::bsu36 ura3::nco met13-2 cyh2-1* |  |
| ACD 166 | ACT 56 | *ade1-1 LYS2 HML HIS4 LEU2 α hoΔ TRP1 ura3::nco met13-2 cyh2-1 sgs1::KANMX4* | WT/ *sgs1Δ* homologous |
|  | ACT 65 | *ade1-1 lys2-c HML::ADE1 HYG-CYH2-his4-r1 leu2-r1-HYG a hoΔ trp1::bsu36 ura3::nco met13-2 cyh2-1 SGS1* |  |
| ADA 1 | Y55 3567 | *ade1-1 LYS2 HML HIS4 LEU2 α hoΔ TRP1 ura3::nco met13-2 cyh2-1 KANMX6::pCLB2-SGS1* | *pCLB2-SGS1*/*sgs1Δ* homologous |
|  | ACT 66 | *ade1-1 lys2-c HML::ADE1 HYG-CYH2-his4-r1 leu2-r1-HYG a hoΔ trp1::bsu36 ura3::nco met13-2 cyh2-1 sgs1::KANMX4* |  |
| ADA 2 | Y55 3565 | *ade1-1 LYS2 HML HIS4 LEU2 α hoΔ TRP1 ura3::nco met13-4 cyh2-1 KANMX6::pCLB2-SGS1 kar1∆13* | *pCLB2-SGS1*/*sgs1Δ* homeologous |
|  | ACT 66 | *ade1-1 lys2-c HML::ADE1 HYG-CYH2-his4-r1 leu2-r1-HYG a hoΔ trp1::bsu36 ura3::nco met13-2 cyh2-1 sgs1::KANMX4 KAR1* |  |
| ADA 3 | ACT 83-1 | *ade1-1 LYS2 HML HIS4 LEU2 α hoΔ TRP1 ura3::nco met13-2 cyh2-1 sgs1-∆C795* | *sgs1-∆C795*/*sgs1Δ* homologous |
|  | ACT 66 | *ade1-1 lys2-c HML::ADE1 HYG-CYH2-his4-r1 leu2-r1-HYG a hoΔ trp1::bsu36 ura3::nco met13-2 cyh2-1 sgs1::KANMX4* |  |
| ADA 4 | ACT 56 | *ade1-1 LYS2 HML HIS4 LEU2 α hoΔ TRP1 ura3::nco met13-2 cyh2-1 sgs1::KANMX4 .* | *sgs1-mlh1-id /sgs1Δ* homologous |
|  | Y55 3543 | *ade1-1 lys2-c HML::ADE1 HYG-CYH2-his4-r1 leu2-r1-HYG a hoΔ trp1::bsu36 ura3::nco met13-2 cyh2-1 sgs1- S1383A,F1385A,F1386A* |  |
| ADA 5 | ACT 2 | *ade1-1 LYS2 HML HIS4 LEU2 α hoΔ TRP1 ura3::nco met13-4 cyh2-1 sgs1::KANMX4 kar1∆13* | *sgs1-mlh1-id/sgs1Δ* homeologous |
|  | Y55 3543 | *ade1-1 lys2-c HML::ADE1 HYG-CYH2-his4-r1 leu2-r1-HYG a hoΔ trp1::bsu36 ura3::nco met13-2 cyh2-1 sgs1- S1383A,F1385A,F1386A KAR1* |  |
| ADA 12 | ACT 56 | *ade1-1 LYS2 HML HIS4 LEU2 α hoΔ TRP1 ura3::nco met13-2 cyh2-1 sgs1::KANMX4* | *sgs1-top3-id/sgs1Δ* homologous |
|  | Y55 3541 | *ade1-1 lys2-c HML::ADE1 HYG-CYH2-his4-r1 leu2-r1-HYG a hoΔ trp1::bsu36 ura3::nco met13-2 cyh2-1 sgs1-K4A,P5A,L9A* |  |
| ADA 23 | ACT 53 | *ade1-1 LYS2 HML HIS4 LEU2 α hoΔ TRP1 ura3::nco met13-2 cyh2-1 SGS1 .* | WT/*sgs1Δ* homologous |
|  | ACT 66 | *ade1-1 lys2-c HML::ADE1 HYG-CYH2-his4-r1 leu2-r1-HYG a hoΔ trp1::bsu36 ura3::nco met13-2 cyh2-1 sgs1::KANMX4* |  |
