# Supplementary material for: The Roles of the Saccharomyces cerevisiae RecQ Helicase SGS1 in Meiotic Genome Surveillance
Source: PLoS One. 2010 Nov 9;5(11):e15380. doi: 10.1371/journal.pone.0015380 (PMC2976770; doi:10.1371/journal.pone.0015380)
Supplement: Table S3 — Map distance for intervals along chromosome III for homologous diploids. The distribution of PDs, NPDs and TTs for homologous diploids were compared using the G-test. After correcting for multiple comparisons using the Benjamini-Hochberg correction [86], p-values <0.05 were considered significant. The map distances for both the homologous diploids are shown in Figure 4A . * - significantly different from WT/WT; # - significantly different from sgs1Δ/sgs1Δ; † - significantly different from sgs1-ΔC795/sgs1Δ; ± - significantly different from pCLB2-SGS1/sgs1Δ; § = significantly different from sgs1Δ combined. (DOC) [file pone.0015380.s003.doc]

**Table S3:** Map distance for intervals along chromosome III for homologous diploids

| **Interval** | **Homologous Diploids** | **PD** | **NPD** | **TT** | **Total Number of Four Viable Spore Tetrads** | **Map Distance (cM)** |
| --- | --- | --- | --- | --- | --- | --- |
| ***HML-HIS4*** | WT/WT (ACD 97) | 196 | 20 | 363 | 579 | 41.7 |
|  | *sgs1Δ/ sgs1Δ* (ACD 95) | 85 | 9 | 109 | 203 | 40.1 |
|  | *sgs1-ΔC795/sgs1Δ* (ADA 3) | 84 | 8 | 109 | 201 | 39.1 |
|  | *pCLB2-SGS1/ sgs1Δ* (ADA 1) | 16 | 2 | 31 | 49 | 43.9 |
|  | *sgs1-mlh1-id/ sgs1Δ* (ADA 4) | 213 | 13 | 373 | 599 | 37.6 |
| ***HIS4-LEU2*** | WT/WT (ACD 97) | 404 | 2 | 165 | 571 | 15.5 |
|  | *sgs1Δ/ sgs1Δ* (ACD 95) | 154 | 5 | 43 | 202 | 18.1 * |
|  | *sgs1-ΔC795/sgs1Δ* (ADA 3) | 152 | 3 | 56 | 211 | 17.5 |
|  | *pCLB2-SGS1/ sgs1Δ* (ADA 1) | 38 | 0 | 10 | 48 | 10.4 |
|  | *sgs1-mlh1-id/ sgs1Δ* (ADA 4) | 433 | 1 | 163 | 597 | 14.2 # |
| ***LEU2-MAT*** | WT/WT (ACD 97) | 340 | 15 | 250 | 605 | 28.1 |
|  | *sgs1Δ/ sgs1Δ* (ACD 95) | 124 | 7 | 100 | 231 | 30.7 |
|  | *sgs1-ΔC795/sgs1Δ* (ADA 3) | 122 | 12 | 110 | 244 | 37.3 |
|  | *pCLB2-SGS1/ sgs1Δ* (ADA 1) | 29 | 2 | 21 | 52 | 31.7 |
|  | *sgs1-mlh1-id/ sgs1Δ* (ADA 4) | 381 | 5 | 234 | 620 | 21.2 ≠ † |
