# Supplementary material for: The Roles of the Saccharomyces cerevisiae RecQ Helicase SGS1 in Meiotic Genome Surveillance
Source: PLoS One. 2010 Nov 9;5(11):e15380. doi: 10.1371/journal.pone.0015380 (PMC2976770; doi:10.1371/journal.pone.0015380)
Supplement: Table S4 — Map distance for intervals along chromosome III for homeologous diploids. The distribution of PDs, NPDs and TTs for homeologous diploids were compared using the G-test. After correcting for multiple comparisons using the Benjamini-Hochberg correction [86], p-values <0.05 were considered significant. The map distances for both the homeologous diploids are shown in Figure 4B . a – sgs1Δ combined represents the collective data from the sgs1Δ/sgs1Δ (ACD 96), sgs1-ΔC795/sgs1Δ (ADA 6) and pCLB2-SGS1/sgs1Δ (ADA 2) homeologous crosses. * - significantly different from WT/WT; # - significantly different from sgs1Δ/sgs1Δ; † - significantly different from sgs1-ΔC795/sgs1Δ; ± - significantly different from pCLB2-SGS1/sgs1Δ; § = significantly different from sgs1Δ combined. (DOC) [file pone.0015380.s004.doc]

**Table S4: Map distance for intervals along chromosome III for homeologous diploids**

| **Interval** | **Homeologous Diploids** | **PD** | **NPD** | **TT** | **Total Number of Four Viable Spore Tetrads** | **Map Distance (cM)** |
| --- | --- | --- | --- | --- | --- | --- |
| ***HML-HIS4*** | WT/WT (ACD 94) | 626 | 0 | 4 | 630 | 0.317 |
|  | *sgs1Δ/ sgs1Δ* (ACD 96) | 95 | 0 | 5 | 100 | 2.5 * |
|  | *sgs1-ΔC795/sgs1Δ* (ADA 6) | 141 | 0 | 8 | 149 | 2.7 * |
|  | *pCLB2-SGS1/ sgs1Δ* (ADA 2) | 194 | 0 | 11 | 205 | 2.7 * |
|  | *sgs1Δ* combined ^a^ | 430 | 0 | 24 | 454 | 2.643 * |
|  | *sgs1-mlh1-id/ sgs1Δ* (ADA 5) | 744 | 0 | 13 | 757 | 0.9 ± |
| ***HIS4-LEU2*** | WT/WT (ACD 94) | 630 | 0 | 1 | 631 | 0.079 |
|  | *sgs1Δ/ sgs1Δ* (ACD 96) | 97 | 0 | 3 | 100 | 1.5 |
|  | *sgs1-ΔC795/sgs1Δ* (ADA 6) | 144 | 0 | 3 | 147 | 1 |
|  | *pCLB2-SGS1/ sgs1Δ* (ADA 2) | 202 | 0 | 3 | 205 | 0.7 |
|  | *sgs1Δ* combined ^a^ | 443 | 0 | 9 | 452 | 0.996 * |
|  | *sgs1-mlh1-id/ sgs1Δ* (ADA 5) | 756 | 0 | 2 | 758 | 0.1 |
| ***LEU2-MAT*** | WT/WT (ACD 94) | 611 | 1 | 19 | 631 | 1.981 |
|  | *sgs1Δ/ sgs1Δ* (ACD 96) | 90 | 1 | 9 | 100 | 7.5 * |
|  | *sgs1-ΔC795/sgs1Δ* (ADA 6) | 132 | 2 | 13 | 147 | 8.5 * |
|  | *pCLB2-SGS1/ sgs1Δ* (ADA 2) | 179 | 2 | 24 | 205 | 8.8 * |
|  | *sgs1Δ* combined ^a^ | 401 | 5 | 46 | 452 | 8.407 * |
|  | *sgs1-mlh1-id/ sgs1Δ* (ADA 5) | 728 | 1 | 31 | 760 | 2.4 ≠ ± † |
