# Supplementary material for: The Roles of the Saccharomyces cerevisiae RecQ Helicase SGS1 in Meiotic Genome Surveillance
Source: PLoS One. 2010 Nov 9;5(11):e15380. doi: 10.1371/journal.pone.0015380 (PMC2976770; doi:10.1371/journal.pone.0015380)
Supplement: Table S5 — Meiosis I non-disjunction events in homeologous diploids. Meiosis I non-disjunction events on chromosome III were identified in homeologous diploids as described in Methods and Materials . Frequencies of meiosis I non-disjunction events out of the total number of tetrads dissected were compared using the G-test. After correcting for multiple comparisons using the Benjamini-Hochberg correction [86], p-values <0.05 were considered significant. * - significantly different from WT/WT; # - significantly different from sgs1Δ/sgs1Δ; † - significantly different from sgs1-ΔC795/sgs1Δ. (DOC) [file pone.0015380.s005.doc]

**Table S5: Meiosis I non-disjunction events in homeologous diploids**

| **Homeologous Diploids** | **Meiosis I Non-Disjunction Events** | **Total Number of Tetrads** | **Percentage of Meiosis I Non-Disjunction Events** |
| --- | --- | --- | --- |
| WT /WT (ACD 94) | 120 | 1040 | 11.5% |
| *sgs1Δ / sgs1Δ* (ACD 96) | 5 | 620 | 0.8% * |
| *sgs1-ΔC795/sgs1Δ* (ADA 6) | 23 | 591 | 3.89% * # |
| *pCLB2-SGS1 / sgs1Δ* (ADA 2) | 25 | 539 | 4.6% * # |
| *sgs1-mlh1-id / sgs1Δ* (ADA 5) | 80 | 1140 | 7.0% * # † |
