# Supplementary material for: The Roles of the Saccharomyces cerevisiae RecQ Helicase SGS1 in Meiotic Genome Surveillance
Source: PLoS One. 2010 Nov 9;5(11):e15380. doi: 10.1371/journal.pone.0015380 (PMC2976770; doi:10.1371/journal.pone.0015380)
Supplement: Table S6 — Oligonucleotides used in this study. Bold sequences, as described by Longtine et al [89], are homologous to pA6a-KANMX6-pCLB2-3HA plasmid [69]. Underlined sequences are homologous to the pAG25 (NATMX4) and pAG32 (HYGMX4) plasmid [77]. (DOC) [file pone.0015380.s006.doc]

**Table S6:** Oligonucleotides used in this study

| **Oligonucleotide** | **Sequence (5’ to 3’)** | **Purpose** |
| --- | --- | --- |
| *pCLB2-SGS1* Fwd | GGAAAAAATACAGATTATTGTTGTATATATTTAAAAAATCATACACGTACACACAAGGCGGTA**GAATTCGAGCTCGTTTAAAC** | Insertion of the *CLB2* promoter |
| *pCLB2-SGS1* Rev | GTAAAGTCGCCGTTTCCTTTAACCATTTGTGCTCCCTTCTTAAGTTATGTGACGGCTTCGTCAC**GCACTGAGCAGCGTAATCTG** |  |
| *pCLB2-SGS1* A1 | AATTCCCATGGCTCAAACTG | Checking *pCLB2-SGS1* transformants |
| *pCLB2-SGS1* A4 | TTGAAGGCGGATCACCTCTA |  |
| *pCLB2-SGS1* Seq F1 | TAAGGTGCCTTAGGGGGACT | Sequencing *CLB2* promoter |
| *pCLB2-SGS1* Seq F2 | GAATCTTTCTGGTATTAATTTTGTCC |  |
| *sgs1-top3-id* Fwd | ATAGTTCAGCCGTGCGTTTC | Sequencing for *K4A,P5A,L9A* mutation |
| *sgs1-top3-id* Rev | TTGAAGGCGGATCACCTCTA |  |
| *sgs1-ΔC795::NATMX4* Fwd | CGATTTTTCATTAAGTGATATAGTGAGTAAATCCAATTTATCTCGTACGCTGCAGGTCGAC | To create the *sgs1-ΔC795* mutation |
| *sgs1-ΔC795::NATMX4* Rev | TGTCGTAGTTATAAGTAACACTATTTATTTTTCTACTCTATCGATGAATTCGAGCTCG |  |
| *RRP7-HIS4::HYG-CYH* Fwd | GCTACTACCTCTCTTTTAATCCAAAATTACAATTTTTACGTTACCGTACGCTGCAGGTCGAC | Insertion of *HYG-CYH* cassette upstream of *HIS4* |
| *RRP7-HIS4::HYG-CYH* Rev | CAAAATACAGTCTTGAATGAATAGAGATACACTATGTAATGAATGGGATCCACTAGTTCTAGAGC |  |
| *LEU2-NFS1::HYG* Fwd | GAAAAAGAATTGCACTTTAACATTAATATTGACAAGGAGGAGGCGTACGCTGCAGGTCGAC | Insertion of *HYG* cassette downstream of *LEU2* |
| *LEU2-NFS1::HYG* Rev | TGACAAGGAGGAGGGCACCACACAAAAAGTTAGGTGTAACATCGATGAATTCGAGCTCG |  |
| K2 | TTCAGAAACAACTCTGGCGCA | Internal primers to check for integration of *KANMX4* |
| K3 | CATCCTATGGAACTGCCTCGG |  |
