# Supplementary figures and images for: The Roles of the Saccharomyces cerevisiae RecQ Helicase SGS1 in Meiotic Genome Surveillance
Source: PLoS One. 2010 Nov 9;5(11):e15380. doi: 10.1371/journal.pone.0015380 (PMC2976770; doi:10.1371/journal.pone.0015380)

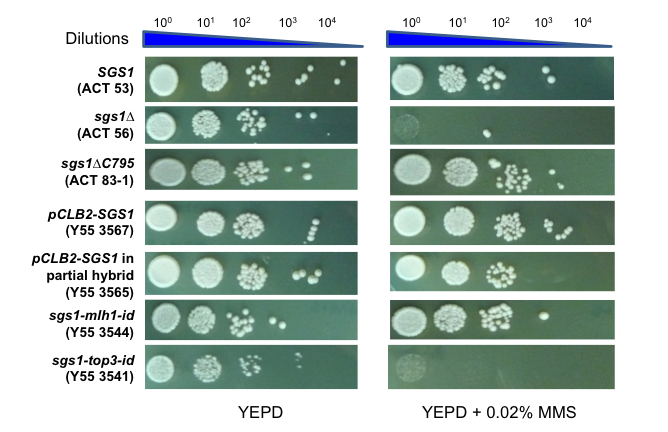

Supplement: Figure S1 — Testing the growth of different sgs1 mutants with respect to MMS resistance by spotting serial dilutions onto YEPD plates (as a control) and YEPD plates supplemented with 0.02% MMS. Failure to grow on YEPD media supplemented with 0.02% MMS is indicative of an inability to repair lesions which lead to the stalling of replication forks during mitosis. (TIFF) [file pone.0015380.s007.tiff]
